# Isolation of five *Enterobacteriaceae* species harbouring *bla*_NDM-1_ and *mcr-1* plasmids from a single paediatric patient

**DOI:** 10.1371/journal.pone.0221960

**Published:** 2019-09-09

**Authors:** F. Martino, N. Tijet, R. Melano, A. Petroni, E. Heinz, D. De Belder, D. Faccone, M. Rapoport, E. Biondi, V. Rodrigo, M. Vazquez, F. Pasteran, N. R. Thomson, A. Corso, S. A. Gomez

**Affiliations:** 1 Servicio Antimicrobianos (National Reference Laboratory on Antimicrobial Resistance), Instituto Nacional de Enfermedades Infecciosas-ANLIS “Dr. Carlos G. Malbrán”, Ciudad Autónoma de Buenos Aires, Argentina; 2 Public Health Ontario Laboratories, Toronto, Ontario, Canada; 3 The Welcome Trust Sanger Institute, Hinxton, Cambridge, United Kingdom; 4 Liverpool School of Tropical Medicine, Liverpool, United Kingdom; 5 Hospital de Niños “Dr. Ricardo Gutiérrez”, Ciudad Autónoma de Buenos Aires, Argentina; 6 London School of Hygiene and Tropical Medicine, London, United Kingdom; Leibniz-Institute DSMZ, GERMANY

## Abstract

In Argentina, NDM metallo-β-lactamase was first reported in 2013. By now, it has disseminated throughout the country in diverse Gram negative bacteria. Here, we report the case of a paediatric patient that underwent a 1-year hospitalisation due to erythrodermic psoriasis in 2014 and received multiple antimicrobial treatments. During his stay, five isolates were obtained from rectal swabs (rs) or blood culture (bc) suspicious of carbapenemase production: a *K*. *quasipneumoniae* subsp. *quasipneumoniae* (rs), *Citrobacter freundii* (rs), *Escherichia coli* (bc), *Enterobacter cloacae* (rs), and a *Serratia marcescens* (bc). The isolates were studied with broth microdilution, biparental conjugation and plasmid and whole genome sequencing (Illumina). All isolates harboured an 138,998-bp type 1 IncC plasmid that carried *bla*_NDM-1_, *ble*_MBL_, *bla*_CMY-6_, *rmtC*, *aac(6’)-Ib*, and *sul1* resistance genes. Additionally, the *bla*_NDM_-plasmids contained IS*Kpn8* an insertion sequence previously described as associated only to *bla*_KPC_. One isolate, a colistin-resistant *E*. *coli*, also carried a *mcr-1*-containing an IncI2 plasmid, which did not harbour additional resistance. The whole genome of *K*. *quasipneumoniae* subsp. *quasipneumoniae* isolate was fully sequenced. This isolate harboured, additionally to *bla*_NDM_, three plasmid-mediated quinolone resistance genes: *qnrB4*, *qnrB52* and *aac(6’)-Ib-cr1*. The *E*. *cloacae* isolate also harboured *qnrA1*. These findings alert to the underestimated horizontal dissemination of multidrug-resistant plasmids limiting treatment options with last resort antimicrobials.

## Introduction

New Delhi metallo-β-lactamase (NDM) is a plasmid-borne carbapenemase that severely limits treatment options against gram-negative pathogens. The *bla*_NDM-1_ gene was initially identified in *Klebsiella pneumoniae* and *Escherichia coli* isolates but later it was reported in many Gram negative species like *Klebsiella oxytoca*, *Proteus mirabilis*, *Enterobacter cloacae*, *Citrobacter freundii*, *Providencia spp*., *Acinetobacter spp*., *Pseudomonas aeruginosa and Vibrio choleae* [1]. Some of these bacteria are carried as gut flora and are commonly found in the environment, providing reservoirs for future infections [2].

The *bla*_NDM-1_ gene is carried on plasmids usually carrying additional resistance genes that compromise antimicrobial treatment. Plasmids associated to the dissemination of *bla*_NDM-1_ vary greatly in attributes such as size, gene content, organization and incompatibility group or replicon type (e.g. IncA, IncC, IncF types, IncL/M, IncN, IncX, and IncH) [3, 4].

The plasmids harbouring *mcr-1* have been identified in multidrug-resistant *Enterobacteriaceae* isolates, some of which co-produced carbapenemases such as KPC, VIM or NDM [5].

Here, we report five *bla*_NDM-1_-producing *Enterobacteriaceae* species isolated from the same patient, one of which co-produced MCR-1. We also characterised the genetic elements that harbour *bla*_NDM-1_ and *mcr-1*, and the high similarities found indicate the presence of one *bla*_NDM-1_ plasmid circulating among these different species.

### Case study

In February 2014, a 4-year-old child suffering from erythrodermic psoriasis with previous hospitalisations was admitted to the hospital due to edematous-ascitic syndrome. After 124 days of hospitalisation the patient died as a result of septic shock. During the patient’s hospitalisation, five *Enterobacteriaceae* species suspicious of carbapenemase production due to imipenem inhibition halos ≤22mm were isolated. As a consequence, he was treated with standard doses of colistin and tigecycline. The timeline of isolation is as follows: at day 39 of hospitalisation, *K*. *pneumoniae* M17277 was isolated from a rectal swab, later identified as *K*. *quasipneumoniae subsp*. *quasipneumoniae*; at day 71, *C*. *freundii* M17394 from a rectal swab; at day 74, *E*. *coli* M17386 from a blood culture; at day 117, *E*. *cloacae* M17464 from a rectal swab and finally, at day 123, *Serratia marcescens* M17468 was obtained from a blood culture. Rectal swabs were periodically obtained as part of the hospital´s surveillance programme to detect KPC-producing *K*. *pneumoniae*. These isolates were sent to the National Reference Laboratory on Antimicrobial Resistance (NRLAR) for further characterisation.

In addition to the clinical situation described above, the patient was also infected and colonized with several microorganisms obtained from rectal swabs, blood cultures or catheter tips as follows: *Staphylococcus aureus*, *Staphylococcus haemolyticus*, *Enterococcus faecalis*, *Enterococcus faecium*, *Acinetobacter baumanii*, *P*. *aeruginosa*, and *Candida parapsilosi*. In addition, *Candida glabrata*, *C*. *albicans* and *Toxocara spp*. were isolated from urine cultures. The patient was treated according to the infecting microorganism with: clindamycin, ceftriaxone, vancomycin, cephalotin, trimethoprim-sulfamethoxazole, colistin, tigecycline, rifampicin, meropenem, minocycline, linezolid, amikacin, gentamicin, ampicillin-sulbactam, teicoplanin, and metronidazol (due to *Giardia lambdia*). In addition, the patient was treated with immunosupressors and antimycotics, as albendazol and fluconazol to treat the mycotic and parasitic infections. Unfortunately, exact dates of isolation of the mentioned isolates are missing in the hospital records.

## Materials and methods

### Microbiological identification, antimicrobial susceptibilities and PCR screening for resistance genes.

Isolates were identified using conventional biochemical tests. All Gram negatives, oxidase negative isolates were tested for *Enterobacteriaceae* species considering lactose fermentation in CLDE media and the following tests: TSI (Triple Sugar Iron), SIM (Sulfide Indole Motility), Citrate media, LIA (Lysine Iron Agar) and MIO (Motility, Indole, Ornithine). These media were purchased in Laboratorios Britania S.A, Argentina. Species identification were confirmed with matrix-assisted laser desorption ionization time of flight (MALDI-TOF) mass spectrometry (Bruker, Germany). Carbapenemase activity was screened with: Triton-Hodge Test, Blue-Carba Test and Carba-NP-Direct test (in house), [6–8]. Screening of metallo-ß-lactamase activity was done by synergy between a meropenem (10 μg) disc and Ethylenediaminetetraacetate acid/Sodium Mercaptoacetate (EDTA/SMA) (Laboratorios Britania S.A., Argentina). Antibiotic susceptibility profiles were determined by agar dilution according to the guidelines and interpretation criteria of the Clinical and Laboratory Standards Institute (CLSI 2018). MICs were determined for imipenem, meropenem, ceftazidime, cefotaxime, cefepime, aztreonam, ciprofloxacin, amikacin, gentamicin, fosfomycin, tigecycline, minocycline and colistin. PCRs were performed using the primers listed in S1A Table to detect the resistance genes: *bla*_PER_, *bla*_CTX-M_, *bla*_KPC_, *bla*_CMY 2/7_, *bla*_VIM_, *bla*_IMP_, *bla*_NDM_, *mcr-1*, *rmtC*, *aac(6’)-Ib-cr* and *qnr*-type (C, D, A, B, S and E) (S1A Table).

### Biparental conjugation

The agar mating method was used to conjugate clinical isolates with E. coli J53 sodium azide-resistant (Az^R^) as recipient [9]. bla_NDM-1_ transconjugants were selected in meropenem (containing 0.2 μg/ml) Luria-Bertani plates plus sodium azide (200 μg/ml). E. coli M17386 harbouring bla_NDM-1_ and mcr-1 was conjugated to a susceptible Salmonella spp. strain M1744 (S1B Table). Transconjugants were identified using Salmonella-Shigella agar plates supplemented with meropenem (0.2 μg/ml) or colistin (2 μg/ml). Subsequently, Salmonella transconjugants producing NDM or MCR were separately conjugated with E. coli J53 Az^R^ as explained above, and the retrotranscojugants were selected as explained above.

### Plasmid characterisation

Plasmid content was analysed by S1 nuclease (Promega, Southampton, UK) digestion followed by PFGE, as described [10]. Plasmids were blotted onto a positively charged nylon membrane (Bio-Rad, Hercules, USA) and hybridised with a bla_NDM-1_ probe (ECL Direct Nucleic Acid Labelling And Detection, Buckinghamshire, England).

bla_NDM-1_- and mcr-1-harbouring plasmids were fully characterised as follows. Plasmids were extracted from transconjugants with Qiagen Large-Construct kit (Qiagen, Hilden, Germany) and sequenced on a MiSeq sequencer with MiSeq Reagent Kit v3 (Illumina, USA). Reads assembly was performed with the CLC Genomics Workbench software (CLC bio, Qiagen) and gaps were closed by PCR using the primers depicted in S1 Table, followed by Sanger sequencing. Open reading frames were predicted and annotated by Prokka v1.12 [11], and manual curation in Artemis [https://www.sanger.ac.uk/science/tools/artemis] and BLAST search [https://blast.ncbi.nlm.nih.gov/Blast.cgi]. ResFinder and PlasmidFinder were used to identify resistance genes and plasmid incompatibility groups, respectively [https://cge.cbs.dtu.dk/services/]. The insertion sequences were identified using ISfinder [https://www-is.biotoul.fr].

### Whole genome sequencing (WGS) and phylogenetic analysis of M17277

Whole bacterial DNA was extracted with QIAcube, using the QIAamp^®^ DNA Mini Kit (Qiagen) and sequenced on a MiSeq sequencer. Reads were *de novo* assembled using VelvetOptimiser v2.2.5 [12]. Automated annotation was done with Prokka [11].

Phylogenetic analysis of M17277 was done with a WGS dataset from 60 type-strains and isolates of *K*. *pneumoniae*, *K*. *quasipneumoniae* subsp. *quasipneumoniae*, *K*. *quasipneumoniae* subsp. *similipneumoniae* and *Klebsiella variicola* (namely, the Kleb-dataset). This dataset comprised: two draft genomes of the type-strains of *K*. *quasipneumoniae* subsp. *quasipneumoniae* and *K*. *quasipneumoniae* subsp. *similipneumoniae*, namely 01A030^T^ and 07A044^T^, respectively [13], and genome assemblies from 58 isolates selected from a collection of 288 isolates previously characterised [14]. These 58 isolates comprised all *K*. *quasipneumoniae* subsp. *quasipneumoniae*, *K*. *quasipneumoniae* subsp. *similipneumoniae* and *K*. *variicola* available in the Holt’s database (1, 19 and 18 isolates, respectively), and 20 isolates randomly selected from the subset of *K*. *pneumoniae* isolates of the Holt´s database (S3 Table) [14]. The multifasta files of the type strains 01A030^T^ and 07A044^T^ were downloaded from NCBI accession numbers CCDF01000000 and CBZR010000000, respectively, and annotated with Prokka [11]. The annotated assemblies of M17277 and the Kleb-dataset were used in a pan-genome analysis with Roary [15] to generate a core-gene alignment (concatenated genes present in ≥99% of the genomes with ≥95% of nucleotide identity). This core-genome alignment was used to generate a single-nucleotide polymorphism (SNP) alignment with SNP-sites v2.3.2 [16] used to construct a maximum likelihood phylogenetic tree with RAxML v8.2.8 [17] under the generalised time reversible model (GTRCAT) and bootstrapping with 1,000 replicates. To support the re-classification of *K*. *quasipneumoniae* subsp. *quasipneumoniae* we used a genome-to-genome distance approach using the online tool at https://ggdc.dsmz.de/ggdc.php [18]

### Nucleotide sequence accession number

Plasmid sequences were submitted to GenBank under accession no. MH995506 (TC-17394), MH995507 (TC17277), MH995508 (TC-17464), MK123267 (RT-17386), MK123268 (TC-17468). K. quasipneumoniae subsp. quasipneumoniae M17277 assembly was submitted under BioProject PRJNA499048 and BioSample: SAMN10340875, GenBank RZJN00000000,

## Results

### Species identification, antimicrobial susceptibilities and resistance genes

The five species were originally identified as *K*. *pneumoniae* M17277, *E*. *coli* M17386, *C*. *freundii* M17394. *E*. *cloacae* M17464 and *S*. *marcescens* M17468. M17277 was one of the first *bla*_NDM-1_-harbouring clinical isolates belonging to the genus *Klebsiella* that had been sent to the NRLAR (2014). *K*. *pneumoniae* M17277 was identified as *K*. *pneumoniae* by biochemical techniques and MALDI-TOF.

The screening tests for carbapenemase activity were positive for all isolates and showed synergy between a carbapenem disc and EDTA/SMA, indicating the production of a metallo-ß-lactamase.

Full resistance phenotype to aminoglycosides (gentamicin and amikacin) was observed in all isolates. Additionally, *K*. *pneumoniae* M17277, *E*. *coli* M17386, *C*. *freundii* M17394, *E*. *cloacae* M17464 were resistant to all β-lactams including carbapenems and aztreonam while *S*. *marcescens* M17468 was susceptible to this last drug (Table 1). *E*. *coli* M17386 was resistant to colistin. Three isolates were resistant to minocycline, two to ciprofloxacin, and two to tigecycline. All isolates remained susceptible to fosfomycin (Table 1).

**Table 1:** Antimicrobial susceptibilities of the clinical isolates anlysed in this study and their respective tranconjugants.

Antimicrobial agent	MIC (μg/ml) / interpretation^a^																								
	KQN-M17277		TC-M17277		ECO-M17386		RT-NDM-M17386*		RT-MCR-M17386*		CFR-M17394		TC-M17394		ECL-M17464		TC -M17464		SMA-M17468^b^		TC-M17468		*E*. *co* J53-Az^R^		
**Imipenem**	16	R	8	R	8	R	8	R	0.12	S	8	R	4	R	32	R	8	R	128	R	32	R	0.06	S	
**Meropenem**	64	R	32	R	32	R	8	R	0.03	S	32	R	16	R	64	R	16	R	64	R	16	R	0.015	S	
**Ceftazidime**	>256	R	>256	R	>256	R	>256	R	0.06	S	>256	R	>256	R	>256	R	>256	R	>256	R	>256	R	0.06	S	
**Cefotaxime**	>256	R	>256	R	>256	R	>256	R	0.03	S	256	R	256	R	>256	R	256	R	>256	R	64	R	0.015	S	
**Cefepime**	256	R	32	R	32	R	16	R	0.06	S	128	R	32	R	128	R	16	R	64	R	16	R	0.06	S	
**Aztreonam**	256	R	4	S	16	R	4	S	0.12	S	8	I	8	I	16	R	4	S	4	S	4	S	0.12	S	
**Ciprofloxacin**	32	R	0.015	S	0.015	S	0.06	S	0.03	S	0.06	S	0.03	S	2	R	0.015	S	0.06	S	0.008	S	0.015	S	
**Amikacin**	>64	R	>64	R	>64	R	64	R	0.5	S	>64	R	>64	R	>64	R	>64	R	>64	R	>64	R	1	S	
**Gentamicin**	>64	R	>64	R	>64	R	>256	R	0.25	S	>64	R	>64	R	>64	R	>64	R	>64	R	>64	R	0.25	S	
**Fosmomycin**^**c**^	16	S	≤16 S		32	S	32	S	32	S	8	S	4	S	32	S	2	S	32	S	2	S	≤ 16	S	
**Tigecycline**^**c**^	16	R	0.5	S	0.5	S	1	S	1	S	0.5	S	0.5	S	16	R	0.5	S	8	R	1	S	≤ 1	S	
**Minocycline**	>64	R	0.5	S	1	S	1	S	0.5	S	0.5	S	0.5	S	16	R	0.12	S	1	S	0.5	S	1	S	
**Colistin**^**c**^	0.25	S	≤1 S		8	R	0.25	S	8	R	0.5	S	≤1	S	0.5	S	≤1	S	ND		N D		≤ 1	S	

Abbreviations used: R, resistant; I, intermediate; S, susceptible; ND, Not determined; TC, transconjugant; RT, retrotransconjugant; KQN, *K*. *quasipneumoniae* subsp. *quasipneumoniae*; ECO, *E*. *coli*, CFR, *C*. *freundii*; ECL, *E*. *cloacae*; SMA, *S*. *marcescens*.

^a^MICs were interpreted according to CLSI 2018 guidelines.

^b^*Serratia* spp. are naturally resistant to colistin.

^c^Fosfomycin, tigecycline and colistin MICs were interpreted following the breakpoints of EUCAST.

*E*. *coli* J53-Az^R^, biparental conjugation acceptor strain, azide resistant.

*RT were obtained from ECO-M17386, RT-NDM-M17386 harbours *bla*_NDM_ plasmid and RT-MCR-M17386 harbours a *bla*_NDM-1_/*mcr* plasmid.

In line with those results, PCR assays showed that all the clinical isolates carried *bla*_NDM-1_ and *rmtC* while *E*. *coli* M17386 additionally carried *mcr-1*. The plasmid mediated quinolone resistance genes PMQRs detected in *K*. *pneumoniae* M17277 were *qnrB4* and *qnrB52* and are described in the text below. *E*. *cloacae* M17464 harboured *qnrA1* which could explain the resistance to ciprofloxacin detected.

### Horizontal transfer of *bla*_NDM-1_ and *mcr-1*-harbouring plasmids

All *bla*_NDM-1_-harbouring plasmids were transferred by conjugation and the susceptibility profiles of transconjugants are shown in Table 1. Together with carbapenem resistance mediated by NDM-1, high level resistance to aminoglycosides was co-transferred mediated by RmtC. Resistance to cephalosporins was transferred mediated by CMY-6. On the contrary, resistance to ciprofloxacin, aztreonam, tigecycline and minocycline was not transferred to transconjugant strains, demonstrating that these resistance determinants may be encoded in the chromosome, in a non-mobile plasmid or in another plasmid that was not transferred.

*E*. *coli-*M17386 harboured both *bla*_NDM-1_ and *mcr-1*. Therefore sequential conjugations were performed to demonstrate that both genes were located on separate conjugative plasmids. In consequence, we obtained two retrotransconjugants that each expressed NDM-1 (RT-NDM-M17386) or MCR-1 (RT-MCR-M17386) (Table 1).

### Whole plasmid characterisation

**Fig 1:**
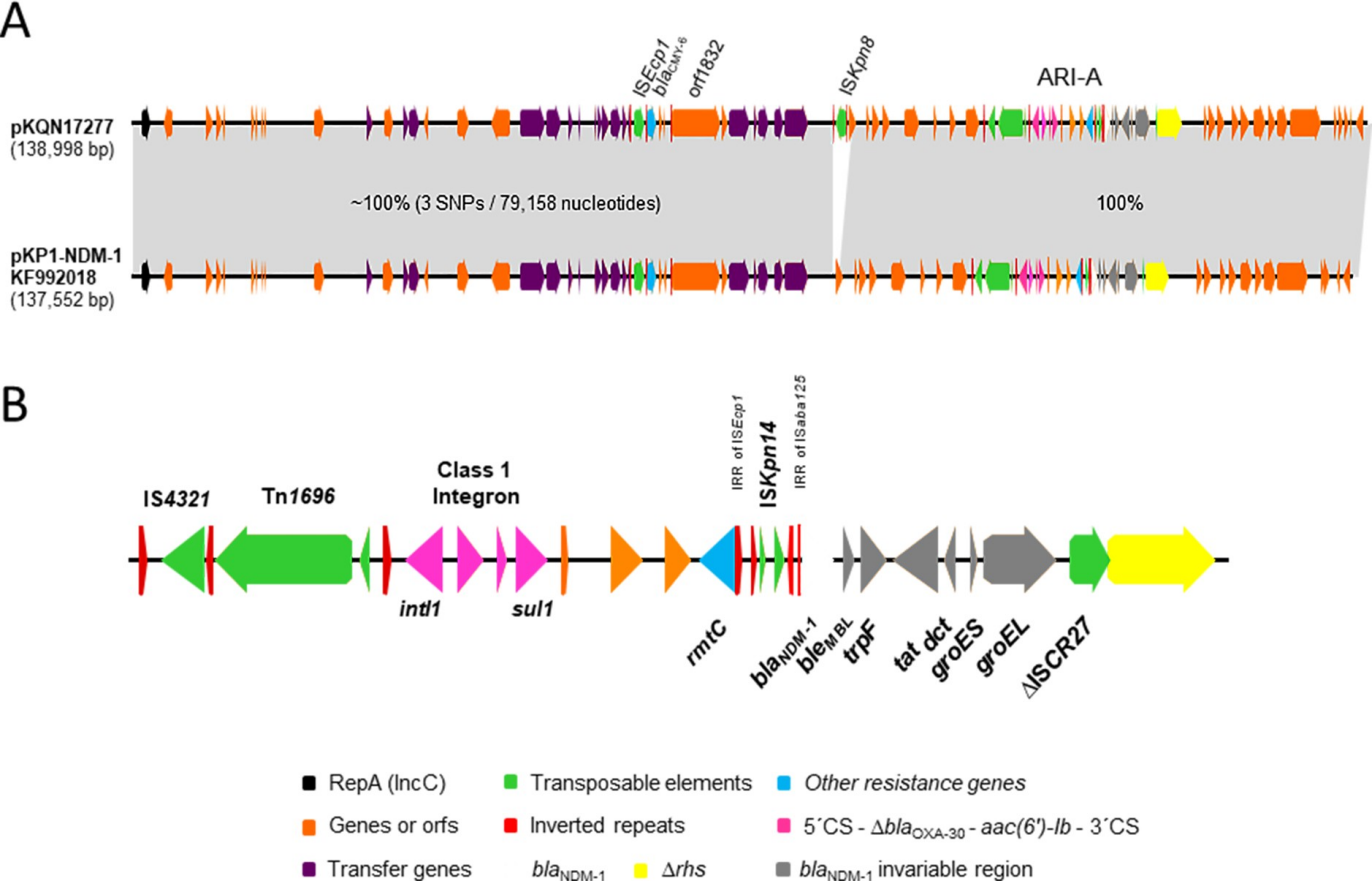
Genetic map of pKQN17277. Genes and orfs are denoted by arrows. Genes, mobile elements and other relevant features are colored as indicated in the key or specified in the figure. Shading denotes regions of identity. A, comparison of the sequenced plasmids and pKP1-NDM-1 (GenBank KF992018). B, main hallmarks of ARI-A resistance island containing *bla*_NDM-1_.

All isolates harboured 2 to 4 plasmids (48.5–470 kb) as observed by S1 nuclease-PFGE (S1A Fig). The *bla*_NDM-1_ probe hybridised with one band of ca. 138-kb in all clinical isolates and their respective transconjugants (S1B Fig). Full sequencing showed that all isolates harboured a plasmid of 138 kb carrying *bla*_NDM-1_. The *bla*_NDM-1_ plasmids from *K*. *pneumoniae* M17277, *E*. *coli* M17386 and *C*. *freundii* M17394, were 100% identical while those from *E*. *cloacae* M17464 and S. *marcescens* M17468 showed 3 and 10 SNPs, respectively, with the former three, and 7 SNPs between them. The plasmid of isolate *K*. *pneumoniae* M17277 (pKQN17277) was selected for the subsequent analysis. pKQN17277 showed maximal identity (99.0%) with pKP1-NDM-1 (acc. no. KF992018) a type 1 IncC obtained from a clinical isolate of *K*. *pneumoniae* in 2010 in Australia from a patient arrived from India [19]. Interestingly, besides 3 SNPs, the unique difference between both plasmids was the presence of the insertion sequence IS*Kpn8* (acc. no. EF382672) in pKQN17277 (Fig 1) not associated to any resistance gene [20]. Moreover, all the NCBI entries that showed 98% cover query and around 99% identity with pKQN17277 did not harbour IS*Kpn8*. Given the high similarity with pKP1-NDM-1, the plasmids characterised here harboured the main hallmarks of type 1 IncC plasmids (Fig 1A and 1B) including *rhs1* (unknown biological function) [21], *orf1832* and ARI-A, which contained *rmtC* and *bla*_NDM-1_ [21] (Fig 1B). This last gene was located within a truncated Tn*125* structure previously reported [4] (Fig 1B). Additionally, these plasmids harboured *bla*_CMY-6_ associated to IS*Ecp1*, which was located outside ARI-A (Fig 1). On the other hand, pKQN17277 was not related to the *bla*_NDM-1_-harbouring plasmid of *K*. *quasipneumoniae* CCBH16302 strain reported in Brazil which was isolated in 2014 (ca. 346 kb, acc.no. MDCA00000000)[22]. Moreover, this last plasmid did not contain *rmtC* and harboured *bla*_NDM-1_ associated with Tn*3000* [17].

Full sequencing of the *mcr-1*-harbouring plasmid of *E*. *coli* M17386 (pMCR-M17386) showed that this gene was located in a ca. 61-kb IncI2 plasmid, which was 100% identical to pMCR-M15224 (acc. no. KY471309) isolated from a clinical isolate of *C*. *amalonaticus* in 2016 from the same paediatric hospital than the case reported here {Faccone, 2018 #4738}[23]. No other antimicrobial resistance gene was identified in pMCR-M17386.

### Phylogenetic analysis of the isolate M17277

**Fig 2:**
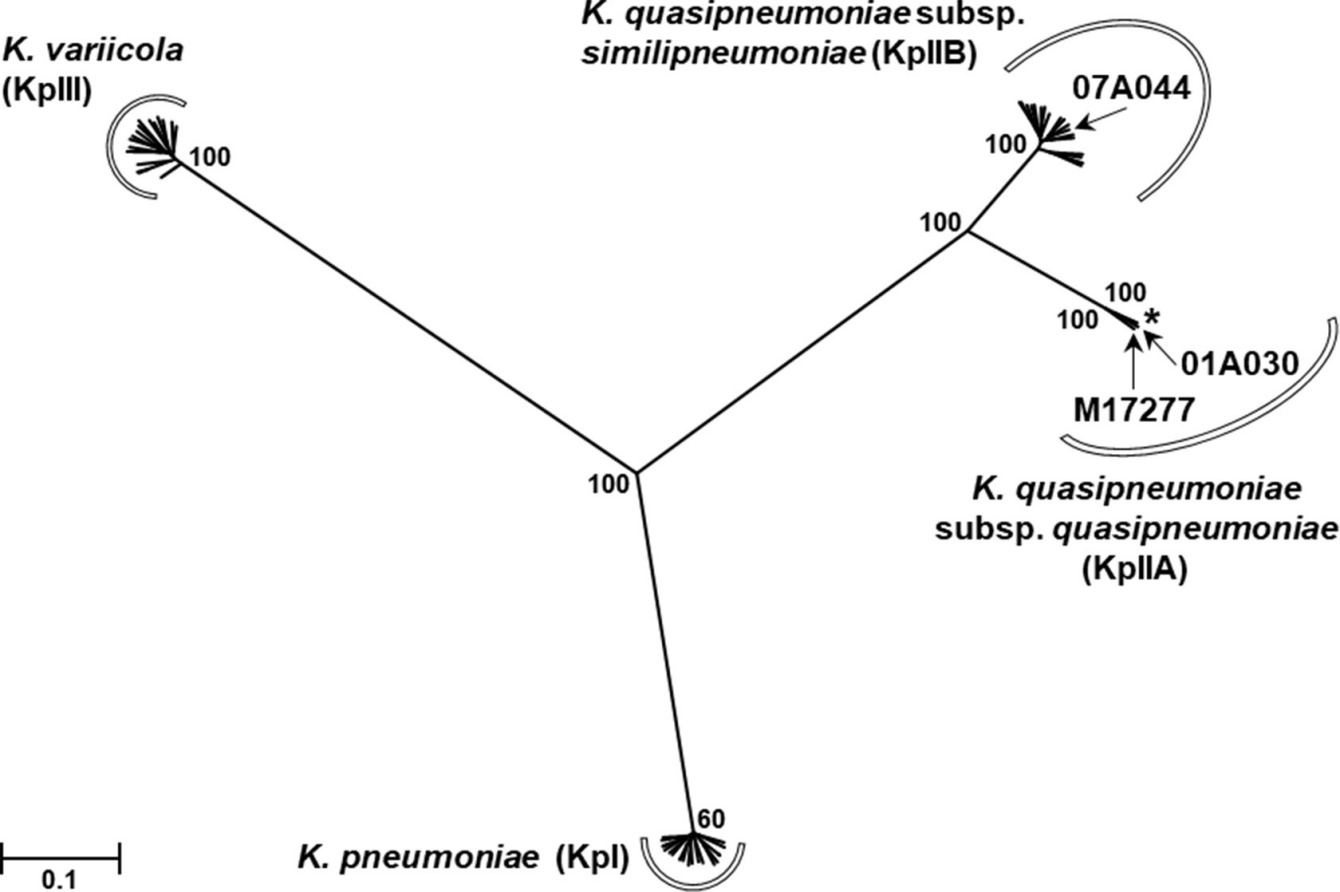
Phylogenetic analysis of the isolate M17277. A maximum likelihood phylogenetic tree was built based on the SNPs in the core-genome assemblies of the isolate M17277 and the Kleb-dataset, which comprised 20 isolates of *K*. *pneumoniae*; 1 isolate of *K*. *quasipneumoniae* subsp. *quasipneumoniae*, plus the type strain 01A030T for this species; 19 isolates of *K*. *quasipneumoniae* subsp. *similipneumoniae*, plus the type strain 07A044T for this species, and 18 isolates of *K*. *variicola*. The final SNP alignment had 61 taxa and 206,622 positions. For simplicity, the taxa names were excluded (arrows indicate the locations of M17277 and both type strains, and the asterisk shows the location of the unique isolate of *K*. *quasipneumoniae* subsp. *quasipneumoniae* of the Kleb-dataset) and the boostrap percentages (over 1,000 replicates) for only relevant nodes are shown. Branch lengths are expressed in units of changes/nucleotide position (scale bar).

M17277 was one of the first *bla*_NDM-1_-harbouring clinical isolates belonging to the genus *Klebsiella* that had been sent to the NRLAR (2014). M17277 was identified as *K*. *pneumoniae* by biochemical techniques and MALDI-TOF. However, these methodologies did not discriminate between *K*. *pneumoniae*, *K*. *quasipneumoniae* and *K*. *variicola* [13]. Therefore, M17277 was subjected to WGS and pan-genome analysis in the context of a global collection (S2 Table) [14]. The phylogenetic tree (S3 Table) showed that the isolate M17277 clustered with 100% bootstrap support with the type strain 01A030^T^ and the unique isolate of *K*. *quasipneumoniae* subsp. *quasipneumoniae* (Fig 2). In order to validate the species identification, we determined the overall genomic similarity between *K*. *quasipneumoniae* subsp. *quasipneumoniae* M17277 and the type strains 01A030^T^ and 07A044 with a genome-to-genome distance calculation. The results confirmed same species between M17277 and 01A030^**T**^ (0.95%G+C) and distinct species between M17277 and 07A044 (1.19% G+C) (see S4 Table). We determined the overall genomic similarity by calculating the genome-to-genome distance between and the type strains 01A030 and 07A044. The results obtained confirmed that M17277 is the same species than the type strain 01A030 with a difference in % G+C: 0.95 and distinct species compared with 07A044 with a difference in % G+C: 1.19 (details in S4 Table).

M17277 showed ciprofloxacin resistance (Table 1). However, the analysis of *gyrA*, *gyrB*, *parC* and *parE*, compared to the corresponding figures of *K*. *quasipneumoniae* subsp. *quasipneumoniae* 01A030^T^, did not show quinolone resistance mutations. Interestingly, M17277 harboured three plasmid-mediated quinolone resistance genes. The *qnrB* alleles *qnrB4* and *qnrB52*, were found in contigs of 14,325 and 2,947 nucleotides, respectively, that showed maximal identity with the plasmids pYNKP001-dfrA (99.98% identity) of *Raoultella ornithinolytic*a YNKP001 and pQnrB52_020046 (100% identity) of *K*. *pneumoniae* SCKP020046, respectively (acc. no. KY270853 and CP028782, respectively). Both *qnrB* alleles were located in the variable region 2 of complex class 1 integrons (associated with IS*CR1*), showing the typical immediate genetic environment of *qnrB* alleles, *i*.*e*., bordered by the genes *sapA* and *pspF* [24]. In addition, *aac(6’)-Ib-cr1* was found in a 3,542-nucleotide contig that encompassed the cassette region of complex class-1 integron [*aac(6’)-Ib-cr1*, *arr-3*, *dfrA27* and *aadA16*], with 100% identity with 26 plasmids from nine enterobacterial species, including *K*. *quasipneumoniae*.

## Discussion

Here, we report the case of a paediatric patient with erythrodermic psoriasis hospitalised due to edematous-ascitic syndrome. The patient was treated with multiple antibiotics including carbapenems and colistin. Notwithstanding the treatments provided, he was infected and colonised with multiple microorganisms including five NDM-1-producing *Enterobacteriaceae*, one co-producing MCR-1. Unfortunately, during 2014, parenteral fosfomycin (all five strains were susceptible to fosfomycin) was not available in that hospital.

The isolate M17277, originally typified as *K*. *pneumoniae*, was found to be *K*. *quasipneumoniae* subsp. *quasipneumoniae*. This species was first proposed in 2014 [13] and, therefore, the current epidemiological information is still scarce [14]. *K*. *pneumoniae* was divided through phylogenetic analysis into three species named *K*. *pneumoniae*, *Klebsiella quasipneumoniae* and *K*. *variicola* [13, 14]. In turn, *K*. *quasipneumoniae* was subdivided into two subspecies, namely, *K*. *quasipneumoniae* subsp. *quasipneumoniae* and *K*. *quasipneumoniae* subsp. *similipneumoniae* (phylogroups KpIIA and KpIIB, respectively) [13, 14]. In 2017, Aires *et*. *al*. reported the first observation of an NDM-1-producing *K*. *quasipneumoniae*, isolated from a rectal swab in Brazil [22]. M17277 was also isolated from a rectal swab and becomes the first report on NDM-1-producing *K*. *quasipneumoniae* subsp. *quasipneumoniae* from Argentina. Besides *bla*_NDM-1_, this isolate also harboured *qnrB4* and *qnrB52* that are epidemiologically relevant since they were not previously reported in Argentina [25]. The presence of these genes, in addition to *aac(6’)-Ib-cr1*, explains, at least in part, the high ciprofloxacin resistance level observed in M17277 (Table 1).

Up to date, there are four reports that involve seven cases of co-carriage of unrelated bacteria with fully sequenced *bla*_NDM_ plasmids [4, 26–28]. In two of these cases, *bla*_NDM_ was located in different plasmids associated with different mobile genetic elements [4, 26]. In other two cases, *bla*_NDM_ was harboured by different plasmids but within the same mobile genetic element [4, 27]. Finally, in the remaining three cases, *bla*_NDM_ was located in identical plasmids (IncC or IncN groups) [4, 28], a situation similar to that reported here. A/C plasmids are now considered IncA and IncC groups [21]. These were the earliest broad host range plasmids to be associated with antibiotic resistance [29]. IncC was divided into type 1 and type 2 plasmids defined by the backbone and resistance island features [29]. Most IncC type 1 plasmids are known to disseminate *bla*_NDM_ within an antibiotic resistance island known as ARI-A [29, 30].

Plasmids highly identical to those found herein were only reported in Australia [19] and USA [31]. Interestingly, the Argentinian plasmids harboured IS*Kpn8*, an insertion sequence that up to date has only been associated to *bla*_KPC_ environment in Argentina and Asia [20]. Moreover, we recently characterised a *C*. *amalonaticus*-producing *bla*_NDM-1_ *and mcr1*.*5* in plasmids highly similar to those reported here, isolated in 2016 and recovered from the same pediatric hospital than the case reported here [32]. Therefore, our analyses contribute to show that *bla*_NDM_ was and continues to be disseminated through several mechanisms worldwide and across species barriers.

The first three *bla*_NDM-1_ plasmids isolated were identical, therefore, it is likely that *K*. *quasipneumoniae-*M17277 colonized the patient and passed pKQN17277 to a colonizing *mcr-1*-harbouring *E*. *coli* strain giving rise to *E*. *coli*-M17386, which later caused a systemic infection. Similarly, *C*. *freundii*-M17394 could have acquired pKQN17277 by conjugation from previously colonizing *Enterobacteriaceae*. The SNPs detected in the *bla*_NDM-1_ plasmids of *E*. *cloacae-*M17464 and *S*. *marcescens-*M17468 suggest independent acquisitions in these isolates, but more data is necessary to corroborate these assumptions.

Colistin is a last resort drugs that, in particular, has been reclassified by the World Health Organization as “Highest Priority Critically Important Antimicrobials” to human medicine, in an attempt to optimize its clinical use to treat serious human infections considering also the emergence of plasmid encoded colistin resistance gene *mcr-1* [33]. In this study, we report an NDM-1-producing *E*. *coli*-M17386 that was additionally resistant to colistin due to the co-expression of MCR*-*1 [23]. The co-production of NDM-type and MCR-type enzymes has already been reported in *E*. *coli* and other *Enterobacteriaceae* species from hospitalized patients [5], healthy and sick food animals like broiler chickens and swine as well as from the farm environment like the slaughterhouse and sewage [34].

## Conclusions

Health institutions worldwide have to affront the lack of effective antimicrobial treatments against multidrug-resistant bacteria and the spread of promiscuous plasmids that are able to disseminate resistance determinants into infecting or colonising unrelated bacterial species. In this case study, we showed the intra-patient dissemination of a *bla*_NDM-1_ harbouring plasmid among different *Enterobacteriaceae* species including the emerging pathogen *K*. *quasipneumoniae* subsp. *quasipneumoniae* [25]. As a consequence, active surveillance measures and strict institutional policies are imperative to determine the local prevalence and prevent further dissemination.

## Supporting information

S1 Fig **(a)Estimation of plasmid content and size of clinical isolates and transconjugants.** Nuclease S1-PFGE of DNA plugs was perfomed to estimate plasmid content and size of the studied isolates. The five clinical isolates and transconjugants harboured between 2 to 4 plasmids (48.5–470 kb). **(b)** S1 nuclease-PFGE and Southern blot with *bla*_NDM-1_ probe of *bla*_NDM-1_ transcojugants
(PDF)

S1 Table (a) List of primers used. (b) *Salmonella spp*. M1744 origin and MIC.
(XLSX)

S2 Table Metadata and accession numbers of the 58 isolates included in the Kleb-dataset selected from the Holt's database [1]. The phylogroups KpI, KpIIA, KpIIB and KpIII correspond to *K*. *pneumoniae*, *K*. *quasipneumoniae* subsp. *quasipneumoniae*, *K*. *quasipneumoniae* subsp. *similipneumoniae* and *K*. *variicola*, respectively.
(XLSX)

S3 Table Stats of the pan-genome analysis of the isolate M17277 and the Kleb-dataset. The alignment of concatenated core genes (highlighted in yellow) resulted in a dataset of 61 taxa with 1,702,827 positions. The subsequent selection of the SNP sites from that core-genome alignment resulted in a SNP alignment of 61 taxa with 206,622 positions, which was used to construct the maximum likelihood phylogenetic tree.
(XLSX)

S4 Table Gene to gene distance results. (XLSX)
