# Correction: Isolation of five *Enterobacteriaceae* species harbouring *bla*_NDM-1_ and *mcr-1* plasmids from a single paediatric patient

**DOI:** 10.1371/journal.pone.0224937

**Published:** 2019-10-31

**Authors:** F. Martino, N. Tijet, R. Melano, A. Petroni, E. Heinz, D. De Belder, D. Faccone, M. Rapoport, E. Biondi, V. Rodrigo, M. Vazquez, F. Pasteran, N. R. Thomson, A. Corso, S. A. Gomez

There is an error in the *bla*_NDM-1_ section of panel B in Fig 1. The authors have provided a corrected version here.

**Fig 1:**
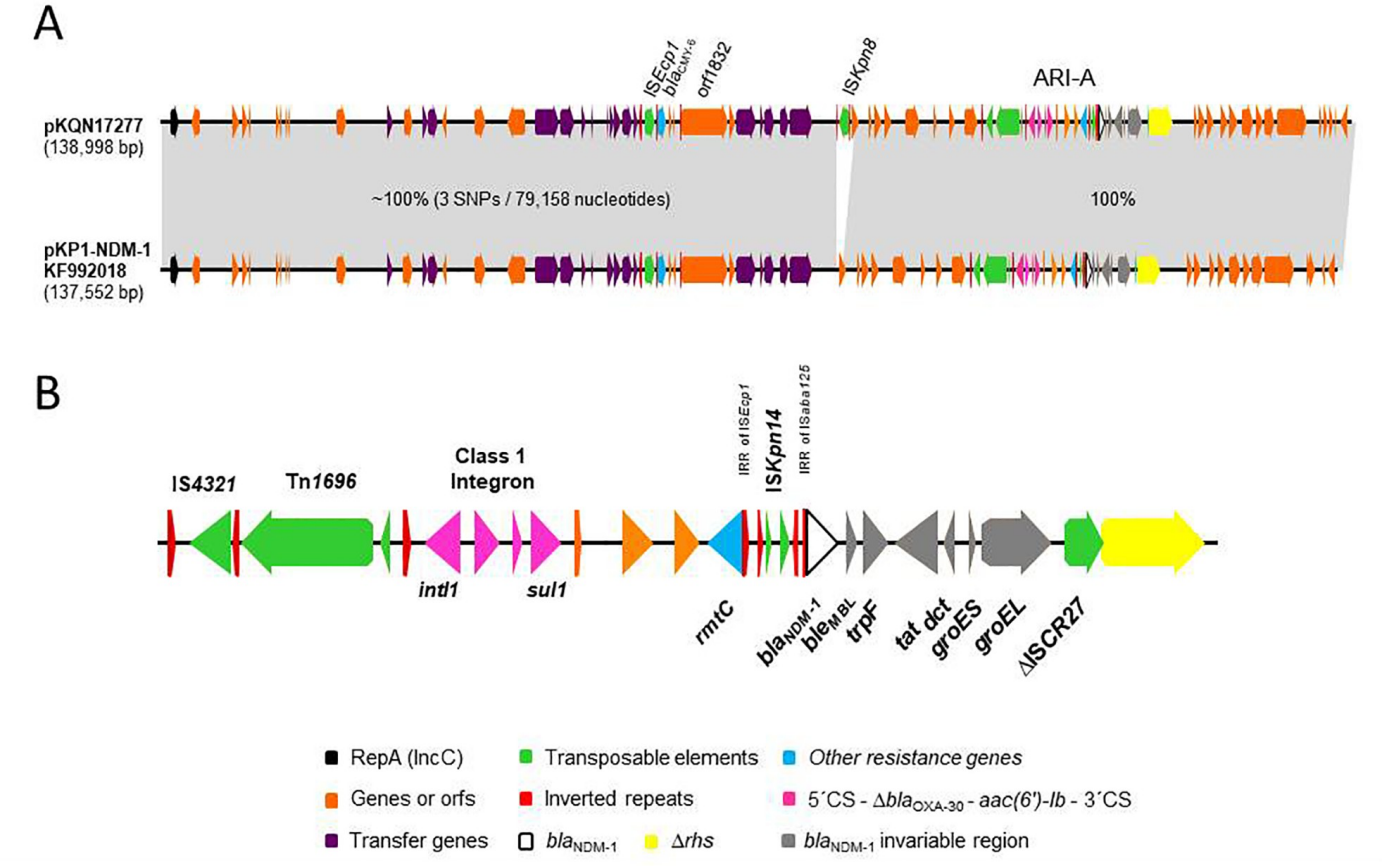
Genetic map of pKQN17277. Genes and orfs are denoted by arrows. Genes, mobile elements and other relevant features are colored as indicated in the key or specified in the figure. Shading denotes regions of identity. A, comparison of the sequenced plasmids and pKP1-NDM-1 (GenBank KF992018). B, main hallmarks of ARI-A resistance island containing *bla*_NDM-1_.
