# Supplementary material for: Isolation of five Enterobacteriaceae species harbouring blaNDM-1 and mcr-1 plasmids from a single paediatric patient
Source: PLoS One. 2019 Sep 9;14(9):e0221960. doi: 10.1371/journal.pone.0221960 (PMC6733481; doi:10.1371/journal.pone.0221960)
Supplement: S1 Fig — (a)Estimation of plasmid content and size of clinical isolates and transconjugants. Nuclease S1-PFGE of DNA plugs was perfomed to estimate plasmid content and size of the studied isolates. The five clinical isolates and transconjugants harboured between 2 to 4 plasmids (48.5–470 kb). (b) S1 nuclease-PFGE and Southern blot with blaNDM-1 probe of blaNDM-1 transcojugants (PDF) [file pone.0221960.s001.pdf]

Figure S1a. Estimation of plasmid content and size of clinical isolates and transconjugants. Nuclease S1-PFGE of DNA plugs was perfomed to estimate plasmid content and size of the studied isolates. The five clinical isolates and transconjugants harboured between 2 to 4 plasmids (48.5-470 kb).

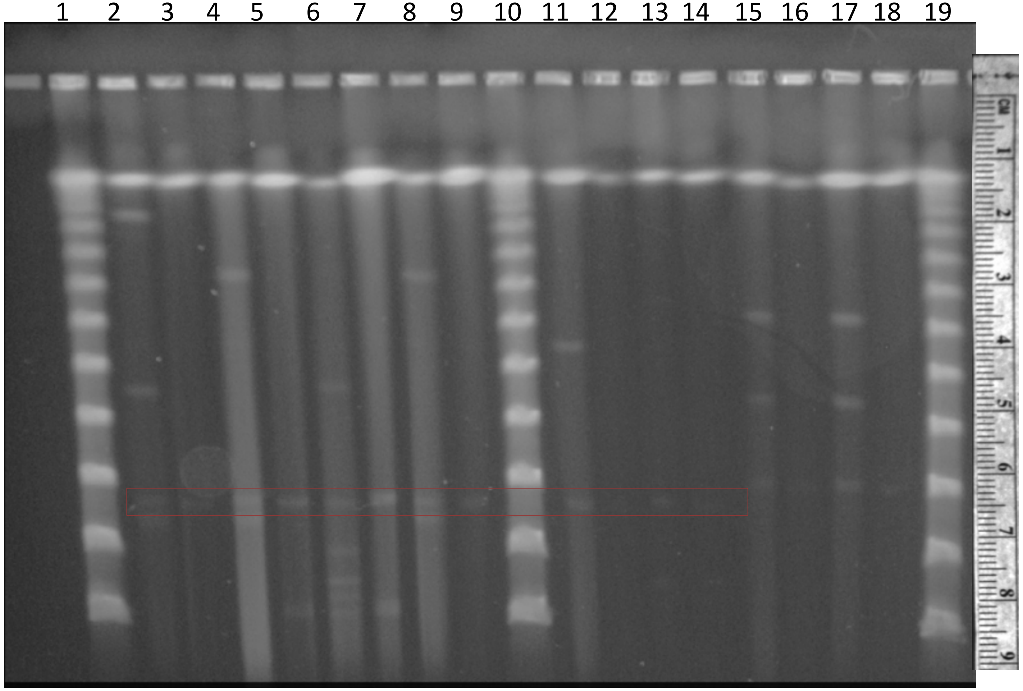

Figure S1. Lane description of the studied isolates is shown in the table below. The red box encloses the bands that hybridized with bla_{NDM-1} probe. Lanes 1, 10 and 19: lambda marker. Lanes 6, 7, 15-18 were not studied here.

LANE	2. KQN-M17277	3. TC-M17277	4. ECO-M17386	5. TC-M17386 *(Salmonella* spp.)	6&7	8. CFR-M17394	9. TC-M17394	11. ECL-M17464	12. TC-M17464	13. SMA-M17468	14. TC-M17468
Aproximate no. of plasmids	4	1	3	2	NOT STUDI ED	3	1	2	1	2	1
Aproximate size (Kb)	470<br>218<br>138<br>115	138	360<br>138<br>115	138<br>48.5		360<br>138<br>115	138	255<br>138	138	138<br>80	138

Abbreviations used: TC, transconjugant; KQN, *K. quasipneumoniae* subsp. *quasipneumoniae*; ECO, *E. coli,* CFR, *C. freundii*; ECL, *E. cloacae*; SMA, *S. marcescens*. Isolates in lanes 6, 7, 15-18 were not studied here.

Figure S1b. S1 nuclease-PFGE and Southern blot with *bla*$_{NDM-1}$ probe of *bla*$_{NDM-1}$ transcojugants

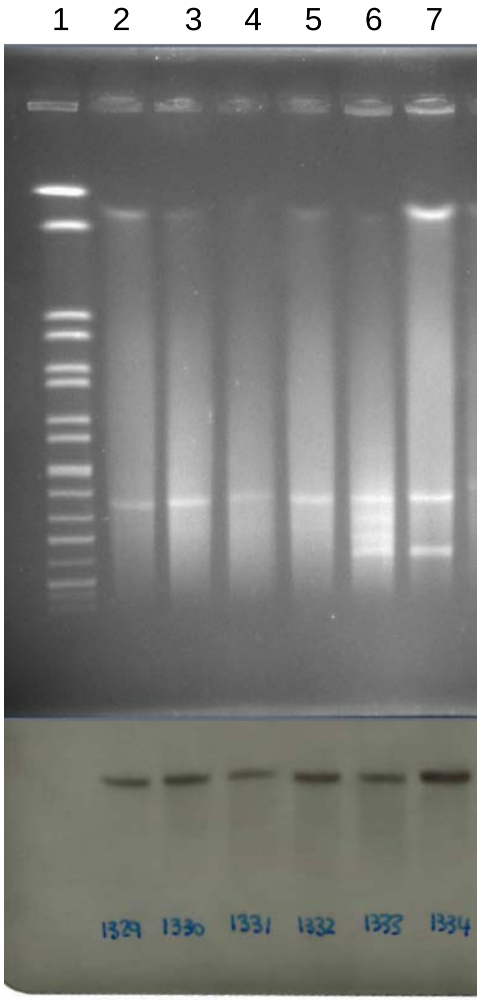

Figure S1b. Plasmids carrying *bla*$_{NDM-1}$ transconjugans were identified by S1 endonuclease digested genomic DNA and PFGE (S1-PFGE) followed by Southern blot analysis using specific probes for *bla*$_{NDM-1.}$ Bands were revealed onto CL-X Posure Film (Thermo Scientific, USA). Above, S1 nuclease-PFGE of from clinical isolates; below, Southern blot hybridization with *bla*$_{NDM-1}$ probe. Below, the *bla*$_{NDM-1}$ probe hybridized with one band of ca. 138-kb in all transconjugants. Lane description: 1. *Salmonella* Ladder; 2, TC-M17277; 3, TC-M17394; 4, TC-M17468; 5, TC-M17464; 6, TC-M17393 (*Salmonella* spp.) not part of this study; 7, TC-M17386 (*Salmonella* spp.); Numbers below in blue ink are internal denominators of each transconjugants.
